# Development of a new method for assessing otolith function in mice using three-dimensional binocular analysis of the otolith-ocular reflex

**DOI:** 10.1038/s41598-021-96596-x

**Published:** 2021-08-25

**Authors:** Shotaro Harada, Takao Imai, Yasumitsu Takimoto, Yumi Ohta, Takashi Sato, Takefumi Kamakura, Noriaki Takeda, Tadashi Kitahara, Makoto Kondo, Yuya Ueno, Shoichi Shimada, Hidenori Inohara

**Affiliations:** 1 Department of Otorhinolaryngology – Head and Neck Surgery, Osaka University Graduate School of Medicine, 2-2 Yamadaoka, Suita, Osaka 565-0871 Japan; 2 Department of Otorhinolaryngology – Head and Neck Surgery, Tokushima University Graduate School of Medicine, Tokushima, Japan; 3 Department of Otorhinolaryngology – Head and Neck Surgery, Nara Medical University, Nara, Japan; 4 Department of Neuroscience and Cell Biology, Osaka University Graduate School of Medicine, Suita, Japan

**Keywords:** Vestibuloocular reflex, Neurophysiology

## Abstract

In the interaural direction, translational linear acceleration is loaded during lateral translational movement and gravitational acceleration is loaded during lateral tilting movement. These two types of acceleration induce eye movements via two kinds of otolith-ocular reflexes to compensate for movement and maintain clear vision: horizontal eye movement during translational movement, and torsional eye movement (torsion) during tilting movement. Although the two types of acceleration cannot be discriminated, the two otolith-ocular reflexes can distinguish them effectively. In the current study, we tested whether lateral-eyed mice exhibit both of these otolith-ocular reflexes. In addition, we propose a new index for assessing the otolith-ocular reflex in mice. During lateral translational movement, mice did not show appropriate horizontal eye movement, but exhibited unnecessary vertical torsion-like eye movement that compensated for the angle between the body axis and gravito-inertial acceleration (GIA; i.e., the sum of gravity and inertial force due to movement) by interpreting GIA as gravity. Using the new index (amplitude of vertical component of eye movement)/(angle between body axis and GIA), the mouse otolith-ocular reflex can be assessed without determining whether the otolith-ocular reflex is induced during translational movement or during tilting movement.

## Introduction

**Figure 1:**
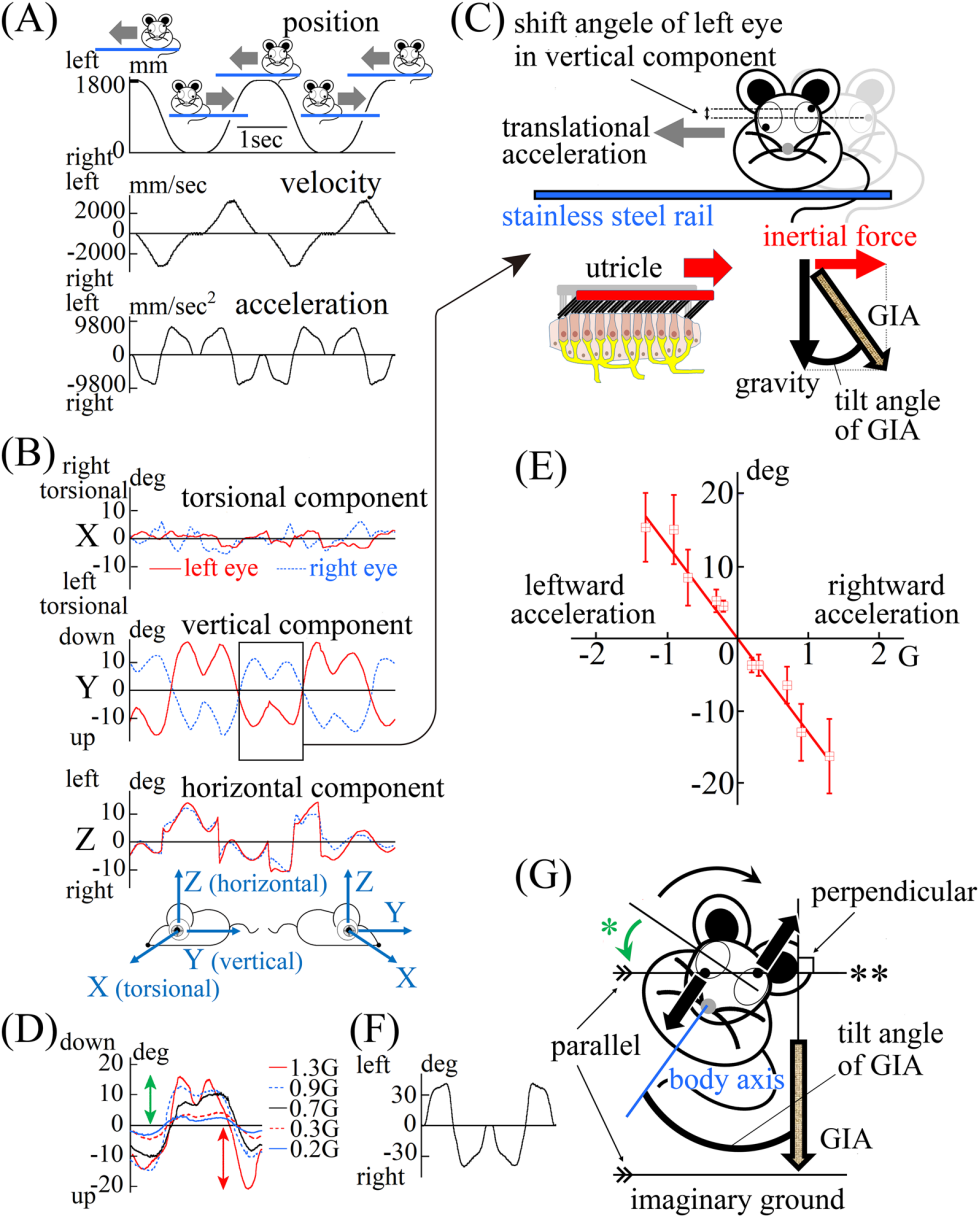
Mice did not exhibit an otolith ocular reflex to compensate for lateral translational motion. (**A**) The change in position of the mouse during lateral translational motion. First column: Mouse position data; Second column: Mouse velocity data; Third column: Mouse acceleration data in the interaural direction. The mouse was reciprocated left and right for a one-way length of 1800 mm in five round trips. The mouse remained at the leftmost and rightmost edges for 0.3 s. The data were collected during the third and fourth trips. The mouse moved at a maximum velocity of 3.25 m/s, and a maximum acceleration of 0.9 G. (**B**) Three-dimensional data from both eyes of a mouse that showed inappropriate disconjugate vertical component during lateral translational motion in light conditions. We recorded movement of both eyes of the mouse using high-speed cameras and analysed the recorded images using an offline computer image analysis system. These data represent the three-dimensional movement of both eyes during the motion shown in (**A**). In the present study, eye movements can be three-dimensionally described by axis angle, which characterizes the eye positions around a single rotation. The three-dimensional coordinates of the eye were defined as follows: the X-axis parallel to the interaural axis (positive left in left eye, positive right in right eye), the Y-axis parallel to the naso-occipital axis (positive backward in left eye, positive forward in right eye), and the Z-axis normal to the X–Y plane (positive upwards) (see insert). The X-, Y-, and Z-components mainly reflect the torsional, vertical, and horizontal components, respectively. The direction of rotation was described from the mouse’s point of view. For the X-component, “right torsional” and “left torsional” indicate that the superior pole of the eyeball rotated to the right and left, respectively. The main component was the vertical component and the waveform (second column) was similar to the waveform of mouse acceleration (third column in (**A**)), not to the waveform of mouse position (first column in (**A**)). The vertical eye movements were disconjugate (i.e., during rightward acceleration, the left eye moved upward and right eye moved downward, and vice versa). The conjugate horizontal eye movement compensating for lateral translational motion observed in humans was not exhibited by the mouse. All mice showed disconjugate vertical eye movement in light conditions. (**C**) Schema showing a mouse exhibiting inappropriate disconjugate vertical eye movement when accelerating rightward. Although the mouse moved laterally on the stainless steel rail, the eyes moved vertically. This eye movement was not able to control gaze appropriately and disturbed the stabilization of gaze in space during the motion. The mouse was loaded with leftward inertia force in the interaural direction. As a result, the mouse was loaded with gravito-inertial acceleration (GIA). When accelerating rightward, the otolithic membrane of the macula of the utricle was moved leftward by inertial force (red arrow). (**D**) During lateral translational motion under dark conditions at five different maximum accelerations, the same vertical eye movement in the mouse’s left eye could be seen in light conditions. The mouse was reciprocated left and right for a one-way distance of 1800 mm in five round trips at five different maximum accelerations under dark conditions, as shown in (**A**). In all mice, the same characteristic eye movement was observed in dark conditions. This graph shows the vertical component of the left eye position data in a mouse during the third trip at each of five different accelerations. The maximum shift angle was proportional to the maximum acceleration. The green double-headed arrow shows the maximum shift angle when the acceleration was 1.3 G. The red double-headed arrow shows the maximum shift angle when the acceleration was − 1.3 G. (**E**) The line created using population-based outcomes of the maximum vertical shift angle data during translational motion in dark conditions. The average maximum shift angle of vertical component of both eyes of all mice during lateral translational motion (**C**,**D**) is set on the ordinate axis, and the maximum linear acceleration is set on the abscissa axis. The maximum shift angle of the vertical component of both eyes was calculated using the following formula: ((maximum shift angle of vertical component of the left eye) − (maximum shift angle of vertical component of the right eye))/2. The maximum shift angle during rightward acceleration (for example, the shift angle shown by the red bidirectional arrow in (**D**)) was plotted on the positive side of the abscissa axis. The maximum shift angle during leftward acceleration (for example, the shift angle shown by the green bidirectional arrow in (**D**)) was plotted on the negative side of the abscissa axis. The error bar shows the 95% confidence interval. The population-based outcomes indicated that the amplitude of the vertical component was proportional to the magnitude of maximum linear acceleration in the interaural direction. (**F**) GIA tilt angle. The GIA tilt angle was calculated using acceleration data at the third trip of five trips when the maximum acceleration was 0.9 G (the first half of the third column in (**A**)) by using the formula: (− 1.0) × tan^−1^ [value of maximum interaural translational linear acceleration/value of gravity acceleration]). The shapes of the waveform are similar among the five different maximum accelerations, although the maximum values of the GIA tilt angle are different. The shape of the waveform was a mirror image relative to the vertical position data of the left eye shown in (**D**). This indicates that left eye vertical movement compensated for the tilt angle of GIA. (**G**) Schema illustrating the mouse’s disconjugate vertical eye movement corresponding to torsion in humans induced by the mouse’s interpretation of GIA as gravity. In the situation of the mouse’s interpretation of GIA as gravity, the imaginary ground is perpendicular to the direction of GIA. During this movement, if the lateral-eyed mouse attempted to set the line passing through the centres of both eyes parallel to the imaginary ground, as in torsion in frontal-eyed humans, disconjugate vertical eye movement would be induced. ** straight line passing through the centres of both pupils. * disconjugate vertical eye movement corresponding to torsion in humans.

**Figure 2:**
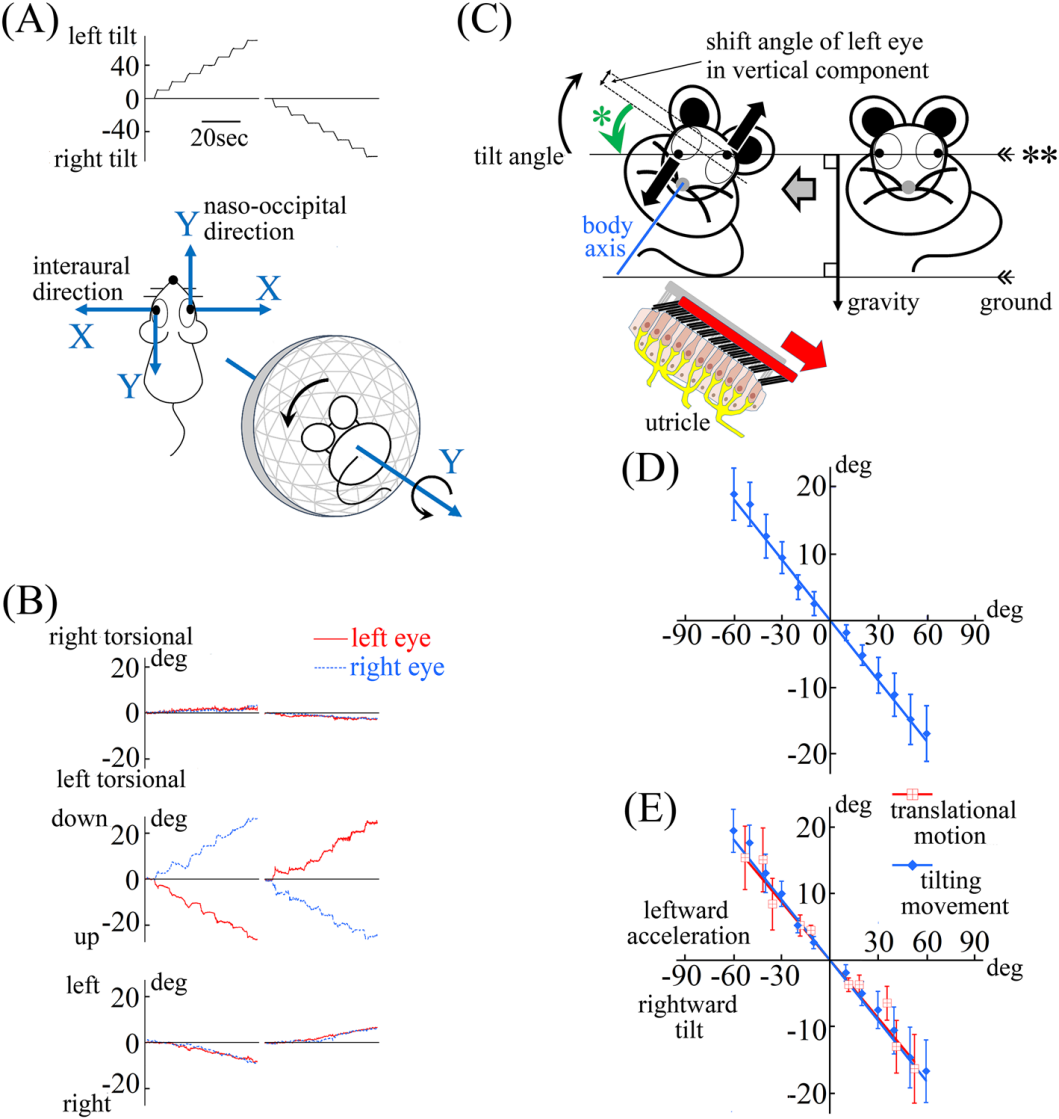
Mice exhibited an appropriate otolith ocular reflex to compensate for lateral tilting movement in dark conditions. (**A**) The tilt angle of the mouse. The mouse was first tilted leftward then tilted rightward. To prevent contamination of the semi-circular canal-ocular reflex, the mouse was tilted as slowly as possible, and remained still for approximately 5 s at each 10° interval. As shown in the inserts, when tilting laterally, the mouse rotates around the Y-axis, whereas the human rotates around the X-axis. Thus, the mouse’s eye movement compensates for the tilting movement with eye movement that rotates around the Y-axis, not around the X-axis. Therefore, the eye movement in the mouse is vertical eye movement, not torsional eye movement, as seen in humans. (**B**) Three-dimensional position data for both eyes of the mouse during lateral tilting movements in dark conditions that compensated for the tilting movements. These data show the three-dimensional positions of the movement of both eyes when tilting in dark conditions, as shown in (**A**). The eye movements were three-dimensionally described in the same way shown in Fig. 1B. As explained in (**A**), both eyes showed no torsional component (first column), but did show a vertical component. As expected from the eye movement results during translational motion (Fig. 1A,B), disconjugate vertical eye movement was observed (second column). When tilting leftward in dark conditions (**A**), both eyes rotated around the Y-axes, the left pupil moved upward, and the right pupil moved downward (second column). Both eyes rotated rightward slightly (third column). Therefore, the line passing through the centres of both pupils (the line indicated by ** in (**C**)) tilts rightward. When tilting rightward, the line tilted leftward because the left pupil moved downward and the right pupil moved upward (second column). Both eyes rotated leftward slightly (third column). All mice showed the same disconjugate vertical eye movements. (**C**) Schema illustrating the mouse’s disconjugate vertical eye movement corresponding to torsion in humans, induced when tilting leftward. The line passing through the centres of both pupils (the line shown by **) is tilted rightward (green curved arrow shown by *). As a result, the line is kept stable in space and almost parallel to the ground by compensating the tilt angle of the mouse. The rotation of the line appears to be similar to torsion in humans. Therefore, the mouse’s disconjugate vertical eye movement appears to correspond to torsion in humans. This disconjugate vertical eye movement is referred to as torsion-like eye movement hereafter. ** straight line passing through the centres of both pupils. * disconjugate vertical eye movement corresponding to torsion in humans. (**D**) The line created using population-based outcomes of the vertical shift angle data during lateral tilting in dark conditions. In the graph, the average shift angle of the vertical component of both eyes of all mice during the lateral tilting movement (**C**) was set on the ordinate axis, and the tilting angles (**C**) were set on the abscissa axis. The shift angle of the vertical component of both eyes was calculated using the formula: ((shift angle of vertical component of the left eye) − (maximum shift angle of vertical component of the right eye))/2. The shift angle during leftward tilt was plotted on the positive side of the abscissa axis. The shift angle during rightward tilt was plotted on the negative side of the abscissa axis. The error bar shows the 95% confidence interval. Population-based outcomes, for all mice, of the averaged shift angle of the vertical component during lateral tilting movement in darkness were proportional to the tilting angle of mice. (**E**) This graph was constructed by overlaying the graphs shown in Fig. 1E and (**D**). To overlay the graphs, the abscissa axis in Fig. 1E was changed from linear acceleration to tilt angle of GIA (see Fig. 1F legend for the method of calculation of tilt angle of GIA from linear acceleration). The data during translational motion (red line) and the data during tilting movement (blue line) were plotted on the same straight line. This result indicates that during both lateral translational motion and lateral tilting movement, the same otolith-ocular reflex (torsion-like eye movement) was exhibited.

The otolith is a sensory organ that responds to linear acceleration. In the interaural direction, linear acceleration is loaded during lateral translational motion, and gravity acceleration is loaded during lateral tilting movement. According to Einstein’s equivalence principle, the two acceleration types cannot be discriminated. The movement of the otolith membrane of the macula of the utricle during lateral translational motion (Fig. 1C) is the same as that during lateral tilting movement (Fig. 2C). However, humans are typically able to discriminate the two acceleration types perceptually, and act in space accordingly. Thus, previous studies have reported that humans use two types of otolith-ocular reflexes^1,2^ that control the gaze to ensure stable perception of the environment during movement^3^. One type of otolith-ocular reflex induces horizontal eye movement during lateral translational motion^1^ and the other type induces torsional eye movement (hereinafter called “torsion”) during lateral tilting movement^2^. Horizontal eye movement and torsion are exhibited even in darkness and are induced by the otolith-ocular reflex as well as by optic flow^1,4^. The appropriate use of two different otolith-ocular reflexes requires higher-order neural pathways combining signals from multiple sensors, including the semi-circular canals and the visual system^5–7^. In the current study, we sought to examine whether lateral-eyed mice possess both types of otolith-ocular reflexes exhibited by species with progressive frontalization of the eyes, including humans and monkeys. Using binocular three-dimensional analysis of the otolith-ocular reflex in mice^8^, the current study revealed that mice only exhibited one type of otolith-ocular reflex, torsion, both during translational linear motion and during tilting movement.

Balance-related falls have been reported to cause more than half of accidental deaths among older people (NIH 1991 Annual Report, National Deafness and Other Communication Disorders Advisory Board). The inner ear is mainly responsible for balance, and the function of the otolith system in the inner ear has been examined in evolutionary studies, clinical studies, and studies of physical principles^9^. In addition, loss of otolith function has been found to lead to patient reports of disorientation and postural unsteadiness^10^. Despite the significant morbidity and mortality associated with loss of otolith function, the pathophysiology of otolith-related diseases remains to be elucidated. A major cause of this lack of research is the absence of an effective functional test of otolith function in mice. The majority of studies of normal and abnormal development of the otolith system have focused on mutant mice^11^. Therefore, the secondary purpose of the current study was to develop an index for evaluating otolith-ocular reflexes in mice, and to identify the normal value of the index, providing a new otolith function test in mice.

## Results

Ten male C57BL/6 J mice at 9–10 weeks of age weighing 20–26 g were used in this study. To induce the otolith-ocular reflex, the otolith was stimulated by linear acceleration using a simple method involving lateral translational linear motion. The mice were reciprocated left and right for a one-way length of 1800 mm in five round trips in light conditions. The profile of the mouse’s position, velocity and acceleration is shown in Fig. 1A, with a maximum velocity of 3.25 m/s and maximum acceleration of 0.9G. During the trips, the movement of both eyes was recorded using 240-Hz high-speed infrared cameras. The data were collected during the third and fourth trips. Figure 1B shows movement data from both eyes of the representative mouse during the lateral translational motion shown in Fig. 1A. Although humans show conjugate horizontal eye movement while reciprocating left and right in light by discriminating between gravity acceleration and translational linear acceleration^12^, mice exhibited disconjugate eye movement, in which the main component was vertical (Y component) (Fig. 1B,C). In addition, the waveform of the vertical component of the eye position data (second column in Fig. 1B) differed from the waveform of the mouse’s position data (first column in Fig. 1A). This eye movement not only failed to stabilize gaze in space during motion but also increased error in the stabilization of gaze. Therefore, this eye movement did not appear to be induced by optic flow. Even in darkness, similar eye movement was observed (attached movie), and the amplitude of the vertical component was proportional to the magnitude of maximum linear acceleration in the interaural direction (Fig. 1D). Figure 1E shows the population-based outcomes, for all mice, of the averaged maximum amplitude of the vertical component calculated using data from both eyes (see figure legend for the calculation method) during lateral translational linear motion when five different maximum acceleration situations (0.2G, 0.3G, 0.7G, 0.9G and 1.3G) in darkness. The amplitude was also proportional to the magnitude of maximum linear acceleration in the interaural direction. These results indicate that eye movement was induced by linear acceleration in the interaural direction (i.e., the otolith-ocular reflex). However, this eye movement is clearly different from that induced by the otolith-ocular reflex in humans. Therefore, we suspected that this eye movement involved the other type of otolith-ocular reflex (i.e., torsion), which occurs during lateral tilting movement in humans. During lateral translational motion, the mouse was not tilted against gravity. The otolith-ocular reflex corresponding to torsion in humans may have been induced in mice during translational motion because the mouse interpreted gravito-inertial acceleration (GIA; i.e., the sum of gravity and inertial force due to translational motion; Fig. 1C) as gravity. We calculated the tilt angle of GIA (Fig. 1C) as shown in Fig. 1F (see figure legend for the method of calculation). The waveforms of vertical eye movement in the left eye were a mirror image of the waveform of GIA tilt angle (Fig. 1D vs F), indicating that vertical eye movement compensated for the GIA tilt angle. This result suggests that the mouse attempted to set a straight line connecting two centres of pupils of both eyes (** in Fig. 1G) to be parallel to the imaginary ground (i.e., perpendicular to the direction of GIA) (Fig. 1G). Therefore, we consider that the lateral-eyed mouse’s disconjugate vertical eye movement (green arrow indicated by * in Fig. 1G) corresponds to torsion in frontal-eyed humans, which suggests that this disconjugate vertical eye movement would be seen during lateral tilting movement.

To verify this hypothesis, we analysed the eye movement of all mice during lateral tilting movement in darkness (Fig. 2A). The frontally positioned eyes of humans rotate around the X-axis (naso-occipital axis) when tilting laterally. Therefore, the torsion that compensates for tilting movement is the torsional eye movement that rotates around the X-axis. However, as shown in Fig. 2A, the laterally positioned eyes of mice rotate around the Y-axis (the naso-occipital axis for mice is the Y-axis, not the X-axis) when tilting laterally. Thus, the mouse’s eye movement compensates for the tilting movement with eye movement that rotates around the Y-axis, not around the X-axis. Therefore, the mouse’s eye movement should be vertical. To prevent contamination of the semi-circular canal-ocular reflex, the mouse was tilted as slowly as possible, and remained still for approximately 5 s at each 10° interval (Fig. 2A). As shown in Fig. 2B, as expected, a representative mouse showed disconjugate vertical eye movement (attached movie) and the straight line connecting the centres of the pupils of both eyes (** in Fig. 2C) rotated in the opposite direction to the mouse’s tilting movement (green curved arrow indicated by * vs. black curved arrow in Fig. 2C). The results revealed that the line was kept parallel to the ground. Figure 2D shows the population-based outcomes, for all mice, of the averaged shift angle of the vertical component calculated using data from both eyes (see figure legend for the method of calculation) during lateral tilting movement in darkness. The shift angle was proportional to the tilting angle of mice. These findings indicate that the otolith-ocular reflex corresponding to torsion in humans (hereafter referred to as torsion-like eye movement) functioned correctly during lateral titling movement (Fig. 2C) and functioned incorrectly during translational motion (Fig. 1C).

To verify whether the same torsion-like eye movement was exhibited both during translational motion and during tilting movement, we compared the two graphs (Figs. 1E and 2D) that were constructed using population-based outcomes. To overlay the two graphs, the abscissa axis in Fig. 1E was changed from linear acceleration to the tilt angle of GIA (see the legend for Fig. 1F for the method of calculation of tilt angle of GIA from linear acceleration). As shown in Fig. 2E, the data during translational motion and the data during tilting movement were plotted on the same straight line. The results indicate that relatively similar torsion-like eye movements were exhibited in mice during both translational motion and tilting movement.

**Figure 3:**
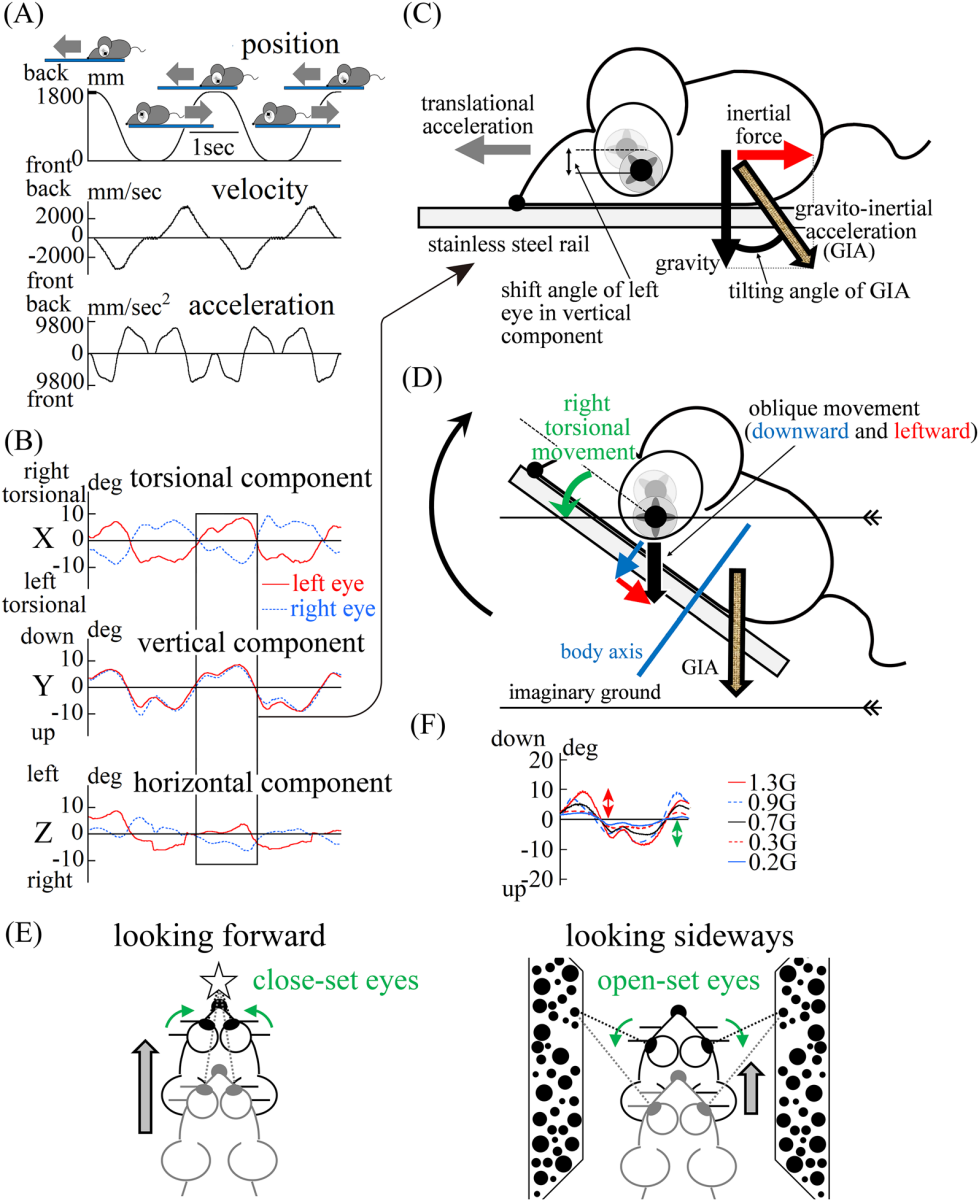
Mice did not exhibit an otolith ocular reflex to compensate for back and forth translational motion. (**A**) The change of mouse position during back and forth translational motion. First column: Mouse position data, Second column: Mouse velocity data, Third column: Mouse acceleration data in the naso-occipital direction. The mouse was reciprocated back and forth for a one-way length of 1800 mm in five round trips. The mouse remained at the leftmost and rightmost edges for 0.3 s. The data were recorded during the third and forth trips. The mouse moved with a maximum velocity of 3.25 m/s, and a maximum acceleration of 0.9 G. The waveforms of position, velocity and acceleration were similar to the waveform shown in Fig. 1A. (**B**) Three-dimensional data from both eyes of a mouse during back and forth translational motion in light conditions. These data are three-dimensional position data for the movement of both eyes during the motion shown in (**A**). The eye movements were three-dimensionally described in the same way shown in Fig. 1B. Disconjugate torsional, conjugate vertical and disconjugate horizontal eye movement were observed. These waveforms had a similar shape to the waveform of the mouse’s acceleration (third column in (**A**)), but were not similar to the waveform of the mouse’s position (first column in (**A**)). This result indicates that eye movement responded to the linear acceleration in the naso-occipital direction and did not compensate the motion of the mouse to stabilize gaze in space. (**C**) Schema of the observed left eye movement in mice that cannot compensate for translational motion in the naso-occipital direction when accelerating forward. When accelerating forward, the left eye showed right torsional and downward movement. This indicates that the mouse did not exhibit an otolith-ocular reflex compensating for translational motion. The mouse was loaded with backward inertia force in the naso-occipital direction. As a result, the mouse was loaded with gravito-inertial acceleration (GIA). (**D**) Schema illustrating the mouse’s left eye movement that compensates for GIA tilt angle by interpretation of GIA as gravity. If the mouse interpreted GIA as gravity, it would attempt to keep the pupil of the left eye still in space, right torsional movement (green curved arrow) would be required to compensate for the tilting movement (black curved arrow), and downward (blue arrow) and leftward (red arrow) movement would be required to compensate for upward head movement against the imaginary ground. Therefore, the left eye movement shown in the boxed part of (**B**) was the eye movement that compensated for the tilting movement of GIA. (**E**) Schema of ideal eye movement when gaze is stabilized in space during forward translational motion. When looking forward during forward translational motion, close-set eye movement should be induced, and when seeing sideways, open-set eye movement should be induced, to stabilize the gaze in space. Eye movements should be disconjugate pure horizontal movements and should not have torsional or vertical components. In reality, eye movements exhibit torsional and vertical components and minor horizontal components as shown in (**B**). Therefore, actual eye-movements are not ideal for stabilizing gaze in space. (**F**) During back and forth translational motion under dark conditions at five different maximum accelerations, the same vertical eye movement in the mouse’s left eye was seen in light conditions. The mouse was reciprocated back and forth for a one-way length of 1800 mm in five round trips at five different maximum accelerations in dark conditions, as shown in (**A**). This graph shows the vertical component of left eye position data of a mouse during the third trip at each of five different accelerations. The maximum shift angle was proportional to the maximum acceleration. This result indicates that these eye movements were induced by the otolith-ocular reflex, not by optic flow during motion. In all mice, the same characteristic eye movement was observed in dark conditions. The green double-headed arrow shows the maximum shift angle when the acceleration was 1.3 G. The red double-headed arrow shows the maximum shift angle when the acceleration was − 1.3 G.

**Figure 4:**
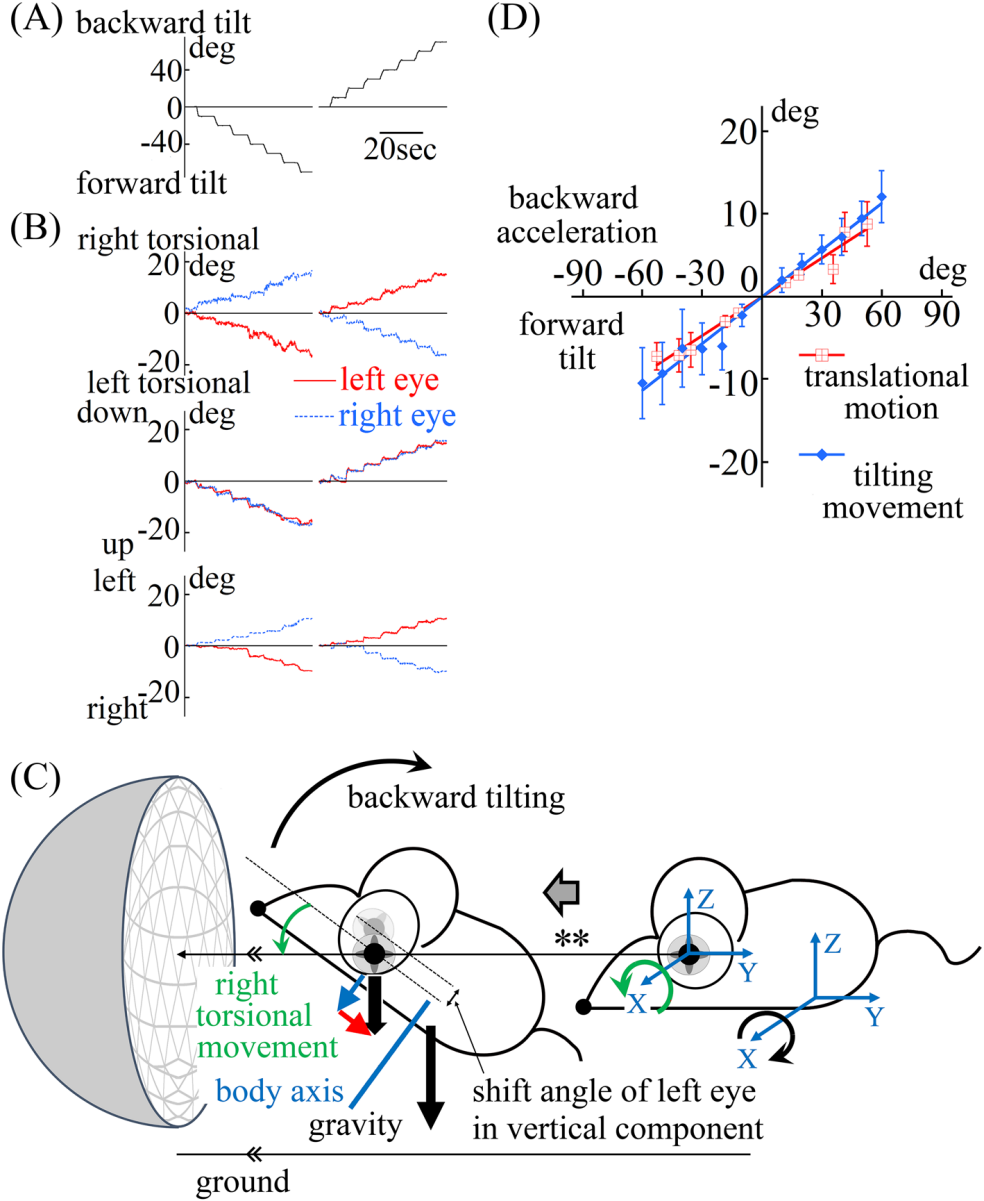
Mice exhibited an appropriate otolith ocular reflex to compensate for forward and backward tilting movement in dark conditions. (**A**) The tilt angle of the mouse. The mouse was first tilted forward then tilted backward. To prevent contamination of the semi-circular canal-ocular reflex, the mouse was tilted as slowly as possible, and remained still for approximately 5 s at each 10° position. (**B**) Three-dimensional position data of both eyes of the mouse during forward and backward tilting movement in dark conditions that compensated for the tilting movement. These data are three-dimensional movement data of both eyes when tilting in dark conditions, as shown in (**A**). The eye movements were three-dimensionally described in the same way shown in Fig. 1B. As expected from the eye movement results during back and forth translational motion (Fig. 3B,D), disconjugate torsional, conjugate vertical and disconjugate horizontal eye movements could be seen. When tilting forward in dark conditions, the left eye showed left torsional movement and the right eye showed right torsional movement to compensate for the tilting movement. The left eye showed upward and rightward movement and the right eye showed upward and leftward movement to compensate the downward head movement. When tilting backward in dark conditions, the left eye showed right torsional movement and the right eye showed left torsional movement to compensate for the tilting movement. The left eye showed downward and leftward movement and the right eye showed downward and rightward movement to compensate for the upward head movement. All mice showed the same disconjugate torsional, conjugate vertical and disconjugate horizontal eye movements. (**C**) Schema illustrating the mouse’s observed left eye movement when tilting backward. Backward tilting movement induces upward head movement. Therefore, to stabilize gaze in space (**) during tilting backward, not only torsional eye movement but also oblique eye movement are needed. The left eye compensated for the tilting movement (black curved arrow) by right torsional movement (green curved arrow) and compensated for the upward head movement by downward (blue arrow) and leftward (red arrow) eye movement. ** Pupil position is stable in space during tilting. (**D**) The line made by using the maximum vertical shift angle data during back and forth translational motion in dark conditions was the same as the line made by using the vertical shift angle data during forward and backward tilting in dark conditions in all mice. The average maximum shift angle of vertical component of the left eye of the all mice during back and forth translational motion (Fig. 3D,F) was set on the ordinate axis, and the maximum tilting angle of gravito-inertial acceleration (GIA) from gravity (Fig. 3D, (− 1.0) × tan^−1^[value of maximum naso-occipital translational linear acceleration/value of gravity acceleration]) was set on the abscissa axis. The maximum shift angle of vertical component of both eyes was calculated using the following formula: ((maximum shift angle of vertical component of the left eye) + (maximum shift angle of vertical component of the right eye))/2. The maximum shift angle during forward acceleration (for example, the shift angle shown by red bidirectional arrow in Fig. 3F) was plotted on the positive side of the abscissa axis. The maximum shift angle during backward acceleration (for example, the shift angle shown by green bidirectional arrow in Fig. 3F) was plotted on the negative side of the abscissa axis. Red squares were plotted and a red approximate straight line was generated. On the graph, the average shift angle of the vertical component of the left eye of the all mice during the forward and backward tilting movement (**C**) was set on the ordinate axis, and the tilting angles were set on the abscissa axis. The shift angle of the vertical component of both eyes was calculated using the following formula: ((shift angle of vertical component of the left eye) + (maximum shift angle of vertical component of the right eye))/2. The shift angle during forward tilt was plotted on the negative side of the abscissa axis. The maximum shift angle during backward tilt was plotted on the positive side of the abscissa axis. Blue rhomboids were plotted and a blue approximate straight line was generated. The error bar shows the 95% confidence interval. The red line and blue lines were almost identical. This result indicates that during both back and forth translational motion and forward and backward tilting movement, the same otolith-ocular reflex that compensated for the tilting movement was exhibited.

To test this possibility further, we investigated the eye movements of all mice when loaded with linear acceleration in a direction other than the interaural direction (i.e., the naso-occipital direction) during back and forth translational linear motion and during forward and backward tilting movement. As expected, during motion in light conditions, disconjugate torsional, conjugate vertical and disconjugate horizontal eye movement compensated for the tilt of GIA (Fig. 3A–D) and did not compensate for translational motion to stabilize gaze in space; if a mouse’s eye movement compensated for motion, the eye movement would be disconjugate pure horizontal eye movement, both when looking forward and when looking sideways, as shown in Fig. 3E. In dark conditions, the same eye movement was observed in a representative mouse (Fig. 3F) (the population-based outcomes of data during motion in darkness are shown later in Fig. 4D).

During forward and backward tilting movement in dark conditions, the same disconjugate torsional, conjugate vertical and disconjugate horizontal eye movement corresponding to torsion-like eye movement was observed (Fig. 4A–C). As shown in Fig. 4D, the line that was constructed using population-based outcomes of the average vertical shift angle data, calculated using data from both eyes (see the Fig. 4D legend for the method of calculation) of all mice during back and forth translational motion in dark (red line), was the same as the line constructed using the population-based outcomes of the average vertical shift angle data calculated using data from both eyes (Fig. 4B,C) (see the Fig. 4D legend for the method of calculation) during forward and backward tilting movement in dark conditions (blue line). These results indicate that the mouse exhibited only one type of otolith-ocular reflex, torsion-like eye movement, when linear acceleration was loaded in the naso-occipital direction and did not exhibit any otolith-ocular reflex to compensate for translational motion (Fig. 3C vs E).

These findings revealed that mice exhibited inappropriate eye movement during translational motion, using only torsion-like eye movement, suggesting that mice have not evolved two types of otolith-ocular reflexes.

## Discussion

Using binocular three-dimensional eye movement analysis, the current study indicated that mice have not evolved two types of otolith-ocular reflexes. Rather, the findings suggested that lateral-eyed animals, such as mice, possess only a primitive otolith-ocular reflex that compensates for tilting movement: torsion-like eye movement (green curved arrow indicated by * in Fig. 2C). The ancestor of modern primates, frontal-eyed shoshonius, evolved from oldest primates, lateral-eyed purgatorius^13^. The progressive frontalization of the eyes resulted in overlap of the left and right visual fields, which led to stereopsis that enabled the perception of depth in the overlapped narrow visual field^14^. In addition, because species with frontalization of the eyes acquired fovea, to which optimal focus is confined, there was a need to direct the optic axis to targets^15^. Because clear vision during translational motion in humans and monkeys requires stabilization of gaze in space via eye movement to compensate for translational motion, the primitive otolith-ocular reflex evolved into an additional type of otolith-ocular reflex that compensates for translational motion by combining signals from multiple sensory pathways, such as semi-circular canals and the visual system^5–7^. As a result, the primitive otolith-ocular reflex (torsion-like eye movement) degenerated and became vestigial in humans and monkeys. Evidence from previous studies suggests that torsion in humans and monkeys cannot compensate for tilting movement because the ratio of torsional angle of the eye (the angle shown by the green curved arrow indicated by * in Fig. 2C) against the head tilting angle (the angle shown by the black curved arrow in Fig. 2C) (torsion gain) is smaller than that in lateral-eyed mice, rats and rabbits^2,16–18^. Without the evolved otolith-ocular reflex, lateral-eyed animals can ensure stable perception of the environment during translational motion because the lateralization of both eyes acquires a whole panoramic field of view. The current results may contribute to a better understanding of the evolution of the otolith-ocular reflex.

In the current study, during translational motion, mice were moved a distance of 1800 mm. If mice attempted to see only one point during the motion, the eye would not have been able to rotate sufficiently to keep seeing the point, and, as a result, the eye position would be saturated. However, if mice did not attempt to see only one point during the motion, the eye position would not be saturated. We speculate that mice in the current study did not attempt to see only one point, for the following reasons. As shown in Figs. 1B and 3B, in all of the components, the eye movement was far from saturation because the amplitude of eye movement was no more than 15° during translational motion. In addition, as shown in the Z component of Fig. 1B, the fast phase of eye movement functioned to avoid the saturation of eye position that was changed by the slow phase of eye movement. First, the current results suggest that the mouse’s otolith-ocular reflex was not correlated with the length of translation during translational motion because the otolith-ocular reflex during translational motion was torsion-like eye movement, not horizontal eye movement. In addition, the mouse’s eye position was dependent on the mouse’s acceleration rather than its position, because, as shown in Figs. 1B and 3B, the waveform of the eye position data was similar to the waveform of the mouse’s acceleration data (third column in Figs. 1A and 3A), not with the waveform of the mouse’s position data (first column in Figs. 1A and 3A). Therefore, even if the length of translation was more than 1800 mm, the mouse’s eye position was not saturated when the mouse’s acceleration was less than the acceleration used in the current study.

In our experiment to measure the otolith-ocular reflex during tilting movement, it was necessary to tilt mice as slowly as possible to prevent contamination of the semi-circular canal-ocular reflex. Oommen and Stahl examined the vestibulo-ocular reflex response to whole-body static tilt in mice while minimizing semicircular canal activation^19^. The results revealed that eye vertical deviation angle was approximately 20° during 60° lateral tilt. This value is similar to the eye vertical deviation angle in the current study during 60° lateral tilt, as shown in Fig. 2D. The eye vertical deviation angle during 60° tilt forward or backward was approximately 15°, and the value was similar to the vertical deviation angle in the current study during 60° tilt forward or backward laterally, as shown in Fig. 4D. These results suggest that the speed of tilt in the current study (2–3 s at each 10° position) was likely to have been sufficient to prevent contamination of the semi-circular canal-ocular reflex. However, regarding whether slow tilting movement is sufficient to prevent contamination, in Oommen and Stahl’s study, mice were only rotated at a slow rate, and the concrete angular velocity of the slow rate was not examined^19^. In addition, we speculate that the current study method successfully eliminated the influence of the semi-circular canal-ocular reflex, because, as shown in Figs. 2B and 4B, the shape of the eye position wave was approximately stepwise, with no overshooting. If a tilt speed of 2–3 s at each 10° position in the current study induced a semi-circular canal-ocular reflex, soon after reaching a static position, the eye position would pass a certain point (overshoot) and go back to the point because the semi-circular canal-ocular reflex induced by the tilting angular velocity is stronger than otolith-ocular reflex induced by gravity acceleration in the interaural or naso-occipital direction. The stepwise shape indicates that during the period in which the mouse was stationary, the eye position was kept still and depended only on the mouse’s tilting angle (i.e., the amplitude of linear acceleration in the intra-aural or naso-occipital direction caused by gravity). Because the semi-circular canal-ocular reflex does not operate while the mouse is stationary, eye movement with a stepwise shape was induced only by the otolith-ocular reflex, which responds to the linear acceleration in the intra-aural or naso-occipital direction.

The secondary purpose of the current study was to develop an appropriate index for evaluation of the otolith-ocular reflex in mice, and to determine the normal value of the index. To the best of our knowledge, the current study is the first to analyse the eye-movements of both eyes in mice three-dimensionally (i.e., with six parameters). As shown in Figs. 1B, 2B, 3B and 4B, regardless of whether eye movement was conjugate or disconjugate, the amplitude of both eyes was the same for each of three components; thus, analysis of either the left or right eye was sufficient. In torsion-like eye movement during lateral translational motion and lateral tilting movement, the vertical component was largest among the torsional, vertical, and horizontal components, and the other components were extremely small, as shown in Figs. 1B and 2B; thus, the vertical component was the optimal parameter under these conditions. In torsion-like eye movement during back and forth translational motion and forward and backward tilting movement, the torsional and vertical components were larger than the horizontal component and the amplitudes of the two components were almost the same as those shown in Figs. 3B and 4B; thus, either the torsional or vertical component is the optimal parameter under these conditions. When analysing eye movement from video-recordings, analysis of the vertical component is easier than analysis of the torsional component because the vertical component can be analysed only by measuring the two-dimensional coordinates of the centre of the pupil in the video. However, to analyse the torsional component, it was necessary to measure the two-dimensional coordinates of both the centre of pupil and an iris freckle in the video (appendix figure). Therefore, the vertical component was the best parameter under these conditions.

During forward and backward tilting movement, as shown in Fig. 4B, large vertical eye movement with the Y axis as the axis of rotation was observed, although mice tilted around the X-axis. During tilting movement, the front of the visual scene moves up and down relative to mice (Fig. 4C) whereas the sides of the visual scene rotate clockwise or counter-clockwise. Therefore, for the stabilization of the fovea image of the sides of the visual scene during tilting movement, torsional eye movement is important (green curved arrow in Fig. 4C), whereas vertical eye movement is important for the stabilization of the fovea image of the front of the visual scene (blue arrow in Fig. 4C). When the mouse’s attention is directed to the front of the visual scene during the tilting movement, large vertical eye movement would be expected to be induced. This may explain why large vertical eye movement was observed during the tilting movement.

Regarding whether light or dark conditions are better for recording the otolith-ocular reflex in mice, the current results indicated that both conditions were acceptable, because the otolith-ocular reflex in dark conditions was similar to that in light conditions (Fig. 1B vs D, Fig. 3B vs F). As above, we propose that the vertical component is the best parameter for evaluating the otolith-ocular reflex. If optic flow induces eye movement in light conditions, the vertical component is not influenced by optic flow because the eye movement induced by the optic flow is purely horizontal. Therefore, when the vertical component is used as an index of the otolith-ocular reflex in mice, either dark or light conditions are acceptable. However, dark conditions may be safer than light conditions because of the possible influence of “crosstalk” on the vertical component. “Crosstalk” refers to diagonal slow-phase eye movement that is evoked during strictly horizontal or vertical optic flow stimuli^20^. Overall, for the analysis of the otolith-ocular reflex in mice, measurement of the vertical component in dark conditions represents the optimal parameter, and the analysis of a single eye is sufficient.

When assessing the otolith-ocular reflex, each of the two types of otolith-ocular reflex have different indexes. When assessing horizontal eye movement during lateral translational motion, the sensitivity of eye movement was assessed using several indexes, such as the values calculated using the following formulae:

- (amplitude of eye movement)/(amplitude of translational motion) (in units of °/cm)^21^, (slow phase eye angular velocity)/(loaded acceleration) (in units of °/s/g), (amplitude of eye movement)/(loaded acceleration) (in units of °/g)^22^, and (amplitude of eye movement)/(amplitude of translational motion)/MA (MA refers to meter-angles, the index of vergence) (in units of °/cm/MA)^23^.

When assessing torsion, the index is the absolute number, the ratio of eye torsional angle against the head tilting angle. When assessing the otolith-ocular reflex in frontal-eyed animals such as humans and monkeys, it is necessary to use different indexes appropriately. However, because lateral-eyed animals such as mice have only one type of otolith-ocular reflex (torsion-like eye movement) one index is sufficient for assessing the otolith-ocular reflex both during tilting movement and during translational motion. During tilting movement, the index is the same as that for frontal-eyed animals (i.e., absolute number, the ratio of amplitude of vertical component of eye movement against head tilting angle (Figs. 2C and 4C). During translational movement, the index is also the absolute number, the ratio of the amplitude of the vertical component of eye movement against the tilting angle of GIA (Figs. 1C,G, 3C,D). These two absolute numbers can be combined into one (i.e., the ratio of amplitude of vertical component of eye movement against the angle between the body axis and GIA). During tilting movement, because GIA refers to gravity itself, the angle between the body axis and GIA is the head tilting angle in space (Figs. 2C and 4C). During translational motion, the angle between the body axis and GIA is the tilt angle of GIA (Figs. 1C,G, 3C,D). On the basis of the above findings, we propose a new index for assessing the otolith-ocular reflex in mice, calculating the absolute number using the following formula:

- (amplitude of vertical component of eye movement)/(angle between body axis and GIA).

Using this index, it is not necessary to determine whether the otolith-ocular reflex is induced by tilting movement or by translational motion, and, as shown in Figs. 2E and 4D, constructed using population-based outcomes, both otolith-ocular reflexes induced by tilting movement and translational motion can be assessed equivalently.

The utricle and saccule are the two organs of the otolith. The left and right utricular maculae are in approximately the same horizontal plane, and, because of this position, are more useful for providing information about the position of the head’s side-to-side tilt when in an upright position^24^. Therefore, the otolith-ocular reflex (i.e., torsion-like eye movement when mice tilt laterally, as shown in Fig. 2C) is induced mainly by the utricle. During lateral translational motion (Fig. 1C), mice showed torsion-like eye movement that responded to the tilt of GIA (Fig. 1G), which was the same as that during lateral tilting movement; thus, the results indicate that the otolith-ocular reflex during lateral translational motion is induced mainly by the utricle. The function of the utricle can be evaluated by calculating our new index during lateral tilting movement (Fig. 2C) or lateral translational motion (Fig. 1G):

- (amplitude of vertical component of eye movement)/(angle between body axis and GIA).

The index reflects the inclination of the straight red and blue lines shown in Fig. 2E. The normal value of the index for assessing the function of the utricle is 0.297 (the average of the two inclinations). In the same way, the saccular maculae are in parallel vertical planes and are likely to respond more to forward and backward tilting of the head^24^; thus, the otolith-ocular reflex during back and forth translational motion is induced mainly by the saccule. Therefore, by calculating our new index, during forward and backward tilting movement (Fig. 4C) or back and forth translational motion (Fig. 3D), the function of the saccule can be evaluated. The index reflects the inclination of the straight red and blue straight lines shown in Fig. 4D. The normal value of the index for assessing the function of the saccule is 0.173 (the average of the two inclinations). The amount of movement of the whole eye during saccule stimulation is thought to be the same as that during utricle stimulation. Therefore, the normal value of the index of the saccule should be the same as that of the utricle. However, the normal value of the index of the saccule (0.173) was lower than that of the utricle (0.297). A possible reason for this difference is as follows. As shown in Figs. 1B and 2B, the main component of eye movement during utricle stimulation is the vertical component, although, as shown in Figs. 3B and 4B, the main component of eye movement during saccule stimulation is divided into two components: torsional and vertical. Therefore, the value of the vertical component of eye movement during utricle stimulation is larger than that during saccule stimulation. As a result, the normal value of the index of the utricle calculated using only the vertical component is larger than that of the saccule calculated using only the vertical component.

One limitation of the current study is that we only examined linear translational acceleration that was perpendicular to the direction of gravity. Although the eye position was determined by the direction of gravity or GIA, we did not observe eye movement when the magnitude of gravity was changed by the stimulation of linear translational acceleration in the same direction as gravity, (i.e., during free-fall).

In conclusion, the current findings demonstrated that mice have not evolved two types of otolith-ocular reflexes, and exhibit only one type (torsion-like eye movement). We propose a new index for assessing the otolith-ocular reflex in mice, as follows:

Absolute value:

- (amplitude of vertical component of eye movement)/(angle between body axis and GIA).

The normal value of the index reflecting the function of the utricle is 0.297, and the normal value of the index reflecting the function of the saccule is 0.173.

## Methods

This study was designed on the basis of the ARRIVE guidelines^25^.

### Animals

Ten male C57BL/6 J mice at 9–10 weeks of age (average age: 9 weeks) weighing 20–26 g (average weight: 23 g) were used in this study. In the current study, the number of mice was not determined using a statistical method. Rather, the number was determined as the smallest number to grasp the trend of changes in eye movement in response to changes in linear acceleration. The animals were purchased from Japan SLC Inc. (Hamamatsu, Japan). This study was carried out in strict accordance with the recommendations in the Guide for the Care and Use of Laboratory Animals of the National Institutes of Health. The Osaka University School of Medicine Animal Care and Use Committee approved the protocol of the study (Permit Numbers: 21- 086–0, 27-043-000). All surgery was performed under anaesthesia, and every effort was made to minimize animal suffering and reduce the number of animals used. After the experiments, we euthanized the animals by intraperitoneal injection of sodium pentobarbital (Nembutal; 200 mg/kg body weight).

### Surgical procedure

Mice were anaesthetized with an intraperitoneal injection of a mixture of ketamine (100 mg/kg) and xylazine (10 mg/kg) in conjunction with local anaesthesia (1% lidocaine). We made a small incision in the mouse’s head skin and fixed a small metal plate with a screw hole to the centre of the skull using dental cement (Sun Medical, Shiga, Japan). After surgery, mice were isolated and closely observed for 48 h.

### Stimulation of linear acceleration during translational motion

**Figure 5:**
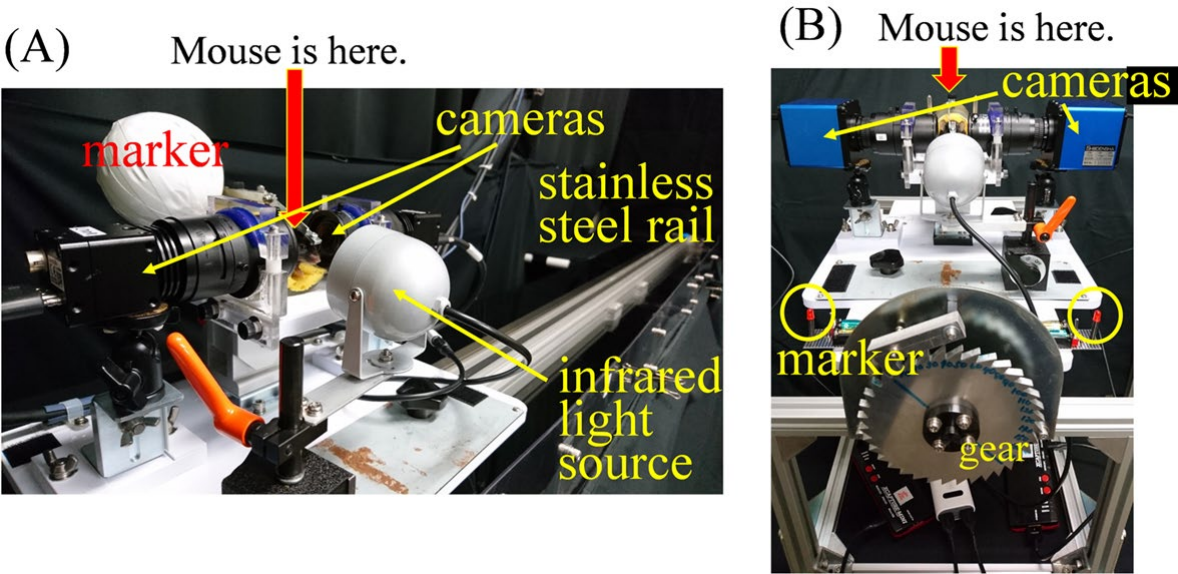
Equipment. (**A**) The stainless steel rail and sled for the stimulation of linear translation. This setting was used for the recording of eye movement during translational motion in dark conditions. Infrared high-speed cameras were set immediately beside the mouse. (**B**) The gear and container for the stimulation of static tilt in dark conditions. Infrared cameras were set immediately beside the mouse.

The mice were subjected to movement in light and dark conditions. A mouse was placed on a linear sled constructed from a plastic cylindrical container and a computer-controlled motor. The mouse was fixed to the sled with a screw by a metal plate attached to the head, and the mouse’s head remained firmly fixed during movement of the sled. The head position was fixed at a position where the bregma-lambda axis was parallel to the ground. The sled moved on a linear stainless steel rail that was parallel to the ground (Figs. 1C, 3C and 5A). The sled and rail were constructed by Bio-Medica Co., Ltd. (Osaka, Japan). From the rightmost edge, the sled was accelerated, then decelerated, and travelled 1800 mm to the leftmost edge. The sled was accelerated, then decelerated, and returned to the rightmost edge. The sled made five round trips. At both the rightmost and leftmost edges, the sled was stationary for approximately 0.3 s. Five settings were used, with maximum acceleration and velocity as follows: 1.3 G (3.25 m/s), 0.9 G (3.25 m/s), 0.7 G (3.06 m/s), 0.3 G (2.24 m/s) and 0.2 G (1.69 m/s). The mouse was placed in the sled in transverse and longitudinal orientations. When transverse, the mouse was moved rightward and leftward, and linear acceleration was loaded in the interaural direction (dark grey arrow in Fig. 1C). When longitudinal, the mouse was moved forward and backward, and linear acceleration was loaded in the naso-occipital direction (dark grey arrow in Fig. 3C). The settings were performed in a random order. When calculating the maximum shift angle of vertical component of both eyes for making Fig. 1E, 2D, the following formula was used:

- ((maximum shift angle of vertical component of the left eye) – (maximum shift angle of vertical component of the right eye))/2

The above value was calculated both during leftward acceleration and during rightward acceleration.

When calculating the maximum shift angle of vertical component of both eyes for making Fig. 4D, the following formula was used:

- ((maximum shift angle of vertical component of the left eye) + (maximum shift angle of vertical component of the right eye))/2

The above value was calculated both during forward acceleration and during backward acceleration.

No adverse events were caused by stimulation via linear acceleration.

### Static tilt

The experiment was performed in dark conditions. The mouse was placed in a plastic cylinder container and fixed to the device with a screw by a metal plate attached to the head. The container was fixed to a board. The board had a gear with 36 teeth positioned at 10° intervals and meshed at 10° (Fig. 5B). The mouse was fixed to the board in two body positions. In one body position, the mouse was rotated laterally about the roll axis (Figs. 2A, 2C, and 5B). In the other body position, the mouse was rotated back and forth about the pitch axis (Y axis) (Fig. 4C). The board was rotated manually, and was held at the 0°, 10°, 20°, 30°, 40°, 50°, 60° and 70° rotated positions for approximately 5 s. The rotated position was changed slowly (3–5°/s) over 2–3 s (Figs. 2A, 4A). The direction of rotation was selected randomly.

When calculating the shift angle of vertical component of both eyes for making Figs. 1E, 2D and 2E, the following formula was used:

- ((shift angle of vertical component of the left eye) – (shift angle of vertical component of the right eye))/2

When calculating the shift angle of vertical component of both eyes for making Fig. 4D, the following formula was used:

- ((shift angle of vertical component of the left eye) + (shift angle of vertical component of the right eye))/2

No adverse events were caused by stimulation via tilting movement.

### Eye movement recording

To record the movement of both eyes during stimulation by translational linear acceleration, a high-speed infrared camera (sampling rate 240 Hz) (STC-CL338A Sentech Co., Ltd, Kanagawa, Japan) was used. The acquisition of images of both eyes was synchronized using a software program (Stream-Pix; NorPix, Montreal, Canada). In light conditions, as a preliminary experiment, we set the angles of the right and left camera axis at 60° to the interaural axis to minimize obstruction of the field of view. During the experiment, we set the angles of the right and left camera axis at 30° to the interaural axis to obtain clear images of the eyes. By comparing the data of the actual experiment and preliminary experiment, we confirmed that the data were similar in both experiments and confirmed that the camera position did not affect eye movement during the movement. In dark conditions, the cameras were set directly beside the eyes (Fig. 5A). Movements of the eyes during the third and fourth trips were analysed.

The infrared camera (sampling rate 60 Hz) (GR200HD2-IR, Shodensha Co., Ltd, Osaka, Japan) was used to record the movement of both eyes during tilting movement in dark conditions. The acquisition of images of both eyes was synchronized using a colour quad processor (SG-202II; Daiwa, Japan). The cameras were set directly beside the eyes (Fig. 5B).

When recording eye movement in darkness, the pupils were contracted with an ophthalmic solution (1% pilocarpine hydrochloride; Nippon Tenganyaku Kenkyusho, Nagoya, Japan).

### Three-dimensional analysis of eye movements

The eye movement images were analysed using an algorithm developed in our laboratory^8,26^ (see Appendix 1). The eye position is represented by a vector around the axis, of which the length is proportional to the angle of rotation. The reference position was defined as the eye position when remaining stationary. The head coordinates for analysing left and right eye movements, as measured by referencing the centre of the pupil and an iris freckle, were reconstructed in three dimensions and defined as shown in Fig. 1B. The X-, Y-, and Z-components mainly reflect the torsional, vertical, and horizontal components, respectively. For the X-component, “right torsional” and “left torsional” indicate that the superior pole of the eyeball rotated to the right and left, respectively. The rotation vector **r** describing a rotation of θ around the axis **n** was given by the formula **r** = **n** tan(θ/2), with **n** being the unit vector, the direction of which represents its axis. The value of the axis angle refers to the Euler angle, and not to tan(θ/2). Accordingly, we used the Euler angle parameter, given as 2 tan^−1^ (magnitude of rotation vector), to represent eye position as an axis-angle representation^27^. Because the camera axis was set at 30° to the interaural axis during translational motion in light conditions, **r** of the left eye was calculated using the following formula.

$${\mathbf{r}} = \left( {\begin{array}{*{20}c} {\cos 30^\circ } & { - \sin 30^\circ } & 0 \\ {\sin 30^\circ } & {\cos 30^\circ } & 0 \\ 0 & 0 & 1 \\ \end{array} } \right)\quad \left( {{\text{analysed}}\,{\mathbf{r}}} \right)$$

**r** of the right eye was calculated using the following formula.

$${\mathbf{r}} = \left( {\begin{array}{*{20}c} {\cos ( - 30^\circ )} & { - \sin ( - 30^\circ )} & 0 \\ {\sin ( - 30^\circ )} & {\cos ( - 30^\circ )} & 0 \\ 0 & 0 & 1 \\ \end{array} } \right)\quad \left( {{\text{analysed}}\,{\mathbf{r}}} \right)$$

In the current study, we measured only eye movement, and did not measure any secondary data in mice.

### Recording and analysis of mouse movement

To record mouse movement during stimulation by translational linear acceleration, the marker was set on the sled (Fig. 5A) and the movement of the marker was recorded using a high-speed infrared camera (STC-CL338A). The acquisition of images of the marker was synchronized with eye images using software (Stream-Pix). The coordinates of the centre of the marker were extracted by binarizing the image of the marker. The position of the mouse was calculated from the coordinate.

To record mouse movement during tilting movement in dark conditions, two markers were set on the board (Fig. 5B) and the movement of the markers was recorded using the infrared camera (GR200HD2-IR). The acquisition of images of the markers was synchronized with images of the eyes using a colour quad processor (SG-202II). The coordinates for the centre of gravity of the two markers were extracted. The tilt angle of the mouse was calculated from the tilting angle of the line connecting the two centres.

## Supplementary Information

Supplementary Information 1.

Supplementary Information 2.

Supplementary Video 1.

Supplementary Video 2.

## Data Availability

We have attached the raw data used to construct Figs. 2E and 4D. In addition, we have attached a movie showing that the mouse exhibited disconjugate vertical eye movement during reciprocating lateral motion at a maximum acceleration of 0.9 G (maximum velocity of 3.25 m/s) in darkness. Data from this movie were used to construct Fig. 1E. We have also attached a movie showing that the mouse exhibited disconjugate vertical eye movement while tilting leftward in darkness. The video is shown at 3 × actual speed. Data from this movie were used to construct Fig. 2D.
