# Supplementary material for: Development of a new method for assessing otolith function in mice using three-dimensional binocular analysis of the otolith-ocular reflex
Source: Sci Rep. 2021 Aug 25;11:17191. doi: 10.1038/s41598-021-96596-x (PMC8387381; doi:10.1038/s41598-021-96596-x)
Supplement: Supplementary file 1 — Supplementary Information 1. [file 41598_2021_96596_MOESM1_ESM.docx]

**Appendix**

***Three-dimensional analysis of eye movements***

In the present study, eye movements were three-dimensionally described by rotation vectors, which characterize the eye positions around a single rotation (Appendix Fig. A). Any eye position can be reached by rotating the eye from the reference position around a single axis. The head coordinates for analysing the left eye were reconstructed in three dimensions and defined as follows: the X-axis was parallel to the interaural axis (positive left), the Y-axis was parallel to the naso-occipital axis (positive backward), and the Z-axis was normal to the X-Y plane (positive upwards) (Appendix Fig. B). The head coordinates for analysing the right eye were reconstructed in three dimensions and defined as follows: the X-axis was parallel to the interaural axis (positive right), the Y-axis was parallel to the naso-occipital axis (positive forward), and the Z-axis was normal to the X-Y plane (positive upwards). Movies of eye movement were converted to 644 × 484-pixel JPEG images and analysed with an algorithm developed in our laboratory (Imai T et al. PLoS One (2016) 11:e0152307). The two-dimensional coordinates of the centre of the pupil (yp zp) and an iris freckle (yi zi) in the image were determined (Appendix Fig. C) (Imai T et al. PLoS One (2016) 11:e0152307). The edge of the pupil was also detected and approximated using an ellipse. We then identified the minor and major axes of the ellipse. The centre of eye rotation (*o*) (yc zc) on the image plane was determined as the intersection of the extensions of the minor axes (Appendix Fig. D) (Imai T et al. PLoS One (2016) 11:e0152307). After determining the centre of eye rotation, we calculated the radius of rotation of the centre of the pupil (*R*) using the following formula: $R\sqrt{1-\left( {the length of minor axis}/{the length of major axis} \right)^{2}}=d$

Here, *d* is the length between *o* and the centre of the pupil ellipse, *p* (Appendix Fig. E). Next, we calculated the length of the radius of rotation of an iris freckle (*R’*). We reconstructed the three-dimensional coordinates of the centre of the pupil and an iris freckle in the head-fixed coordinate system as $\left( \begin{matrix} \sqrt{R^{2}-\left( yp-yc \right)^{2}-\left( zp-zc \right)^{2}} & yp-yc & zp-zc \end{matrix} \right)$ and $\left( \begin{matrix} \sqrt{{R'}^{2}-\left( yi-yc \right)^{2}-\left( zi-zc \right)^{2}} & yi-yc & zi-zc \end{matrix} \right)$.

The relationship between the three-dimensional coordinates of the centre of the pupil and the iris freckle, compared between the test and reference positions, was used to calculate the rotation vector of the eye position, **r** (Haslwanter T. Vision Res (1995) 35:1727-39). The reference position was defined as the eye position when the mouse’s head was still in an upright position. The X, Y, and Z components of the axis angle of the eye position primarily reflected the roll, pitch, and yaw components, respectively (Appendix Fig. B). The direction of rotation was described from the mouse’s point of view. For the X-component, “right torsional” and “left torsional” indicated that the superior pole of the eyeball rotated toward the right and left ears, respectively. We used the Euler angle parameter, given as 2 × tan^-1^(magnitude of rotation vector, **r**), to represent eye position and velocity as an axis-angle representation (Schnabolk C et al. J Neurophysiol (1994) 71:623-638).

The accuracy of this method for analysing eye rotation vectors has been described elsewhere. The accuracy of our video-oculography method was confirmed by matching the analysed angle of an eyeball simulator with a previously set value (Imai et al. Acta Otolaryngol (1999) 119:24-28) and by matching the analysed angle with that analysed using a scleral search coil system in humans (Imai T et al. Auris Nasus Larynx (2005) 32:3-9). In addition, we confirmed the absence of torsional cross-talk between the horizontal and vertical components of the analysed data when the rotated angle was between −40° and 40° (Imai et al. Acta Otolaryngol (1999) 119:24-28).


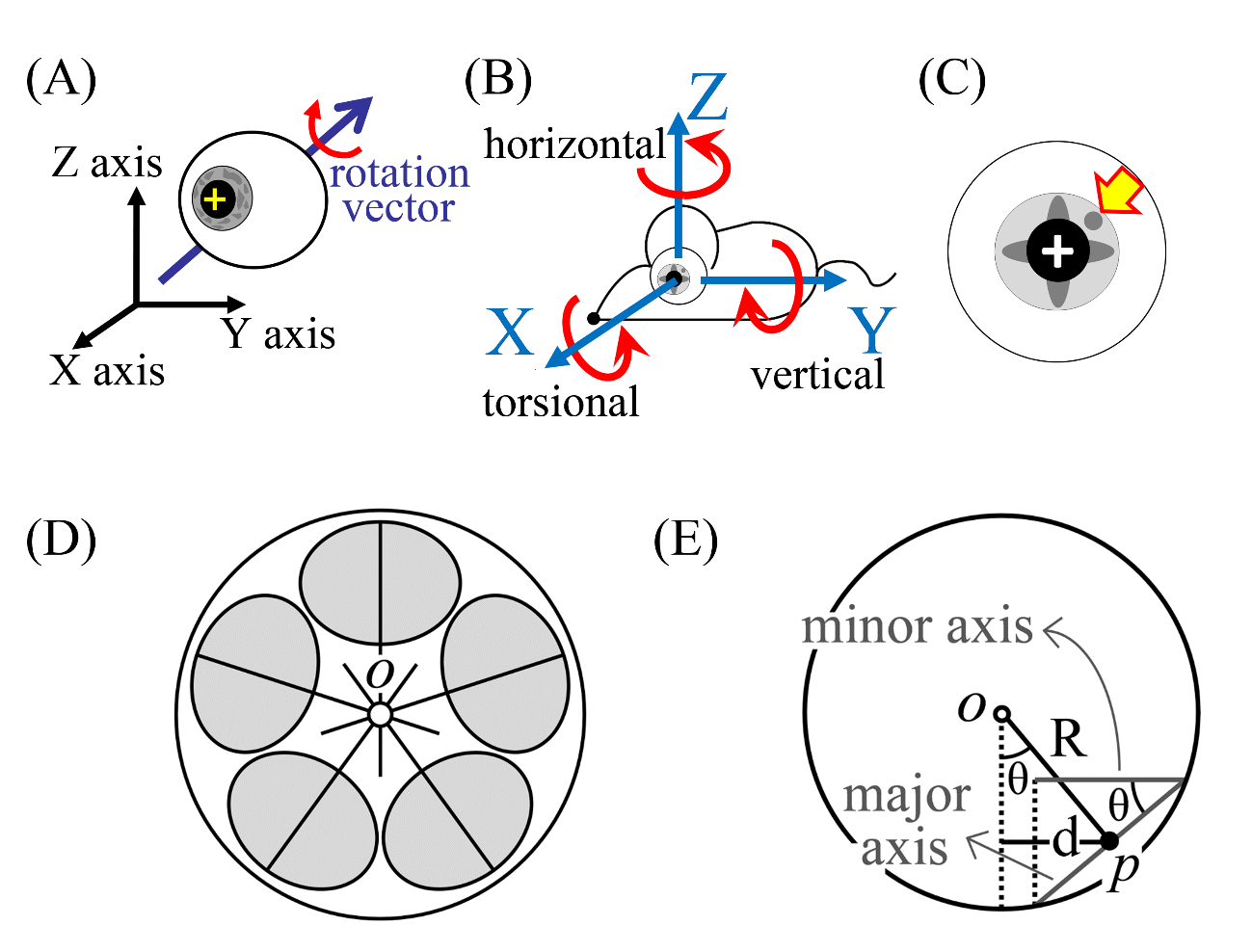


**Appendix figure**

The method for analysing three-dimensional eye position in the mouse

(A) Rotation vector of eye position

Any eye position can be reached by rotating the eye from the reference position around a single axis. Therefore, any eye position can be represented by the axis and the rotation angle around the axis. A rotation vector is a vector of which the direction is the axis and the magnitude is the value of tan(the rotation angle / 2).

(B) Three-dimensional coordinate frame of the left eye

The three-dimensional coordinate of the left eye was defined as follows: the X-axis parallel to the interaural axis (positive left), the Y-axis parallel to the naso-occipital axis (positive backward), and the Z-axis normal to the X–Y plane (positive upwards). In the present study, eye movements can be three-dimensionally described by axis angle, characterizing the eye positions around a single rotation. Therefore, the X-, Y-, and Z-components mainly reflect the roll, pitch, and yaw components, respectively.

(C) The schema of the eye image of the mouse

The coordinates of the centre of the pupil and an iris freckle shown by the arrow were extracted.

(D) The method for determination of the coordinate of centre of eye rotation

The edge of the pupil was detected and approximated using an ellipse. We then identified the minor and major axes of the ellipse. The centre of eye rotation (*o*) on the image plane was determined as the intersection of the extensions of the minor axes.

(E) The method for calculating the radius of rotation of the centre of the pupil

After determining the centre of eye rotation, we calculated the radius of rotation of the centre of the pupil (*R*) using the following formula: $R\sqrt{1-\left( {length of minor axis}/{length of major axis} \right)^{2}}=d$

Here, *d* is the length between the centre of eye rotation, *o* and the centre of the pupil ellipse, *p*.
